# General requirements for the production of extracellular vesicles derived from human stem cells

**DOI:** 10.1111/cpr.13554

**Published:** 2023-09-28

**Authors:** Ling Wang, Shiyu Liu, Ka Li, Aijin Ma, Chenghu Hu, Changlin Wang, Nan Cao, Yunpeng Zhao, Ruifeng Fu, Wenwen Jia, Peng Xiang, Houqi Liu, Zhongquan Qi, Ningwen Zhu, Lingmin Liang, Lei Wang, Jiani Cao, Peijun Zhai, Jiaxi Zhou, Jun Wei, Tao Na, Jun Wu, Zhiying He, Guangdong Zhou, Weifeng Yu, Jinyan Wu, Wen Zeng, Yong Zhang, Lijun Zhu, Boqiang Fu, Jingzhong Zhang, Shuwei Yang, Chengxiang Dai, Hengmi Cui, Jianzhong Jing, Hexin Yan, Xiaowen He, Yongbo Lu, Cailing Tong, Tongbiao Zhao, Jie Hao, Xialin Liu, Yan Jin, Yue Wang

**Affiliations:** ^1^ Department of Stem Cells and Regenerative Medicine Center for Translational Medicine, Naval Medical University Shanghai China; ^2^ Shanghai Key Laboratory of Cell Engineering Naval Medical University Shanghai China; ^3^ State Key Laboratory of Oral and Maxillofacial Reconstruction and Regeneration, National Clinical Research Center for Oral Diseases, Shaanxi Key Laboratory of Stomatology, Department of Prosthodontics School of Stomatology, The Fourth Military Medical University Shaanxi China; ^4^ Institute of Clinical Science Zhongshan Hospital, Fudan University Shanghai China; ^5^ Beijing Technology and Business University Beijing China; ^6^ Chinese Society for Stem Cell Research Shanghai China; ^7^ Xi'an Key Laboratory of Stem Cell and Regenerative Medicine Institute of Medical Research, Northwestern Polytechnical University Shaanxi China; ^8^ China Institute of Standardization Beijing China; ^9^ Center for Stem Cell Biology and Tissue Engineering, Key Laboratory for Stem Cells and Tissue Engineering, Ministry of Education Sun Yat‐Sen University Guangzhou China; ^10^ National Stem Cell Resource Transformation Bank Shanghai China; ^11^ Shanghai Institute of Stem Cell Research and Clinical Translation Shanghai China; ^12^ Haimenshengyuan Stem Cell Technology Co. Ltd. Nantong China; ^13^ Guangxi University Nanning China; ^14^ Fudan University Shanghai China; ^15^ National Stem Cell Resource Center, State Key Laboratory of Stem Cell and Reproductive Biology, Institute of Zoology, Institute for Stem Cell and Regeneration, University of Chinese Academy of Sciences Beijing China; ^16^ Beijing Institute for Stem Cell and Regenerative Medicine Beijing China; ^17^ China National Accreditation Center for Conformity Assessment Beijing China; ^18^ Institute of Hematology and Blood Diseases Hospital, Chinese Academy of Medical Sciences and Peking Union Medical College Tianjin China; ^19^ Zephyrm Biotechnologies Co. Ltd. Beijing China; ^20^ China Institute for Food and Drug Control Beijing China; ^21^ Institute for Regenerative Medicine, Shanghai East Hospital, School of Life Sciences and Technology, Tongji University, Shanghai, China; Shanghai Engineering Research Center of Stem Cells Translational Medicine Shanghai China; ^22^ Shanghai Jiaotong University Shanghai China; ^23^ Army Military Medical University Chongqing China; ^24^ National Clinical Research Center of Respiratory Disease, First Affiliated Hospital of Guangzhou Medical University Guangzhou China; ^25^ HHLIFE Company Inc. Shenzhen China; ^26^ Institute of Scientific and Technical Information of China Beijing China; ^27^ National Institute of Metrology Beijing China; ^28^ Suzhou Institute of Biomedical Engineering Technology Chinese Academy of Sciences Suzhou China; ^29^ Guangzhou Funeng Gene Co. Ltd. Guangzhou China; ^30^ Cellular Biomedicine Group Ltd Shanghai China; ^31^ Jiangsu Innovation Institute For Biomedicine Nanjing China; ^32^ Shanghai Golden Meditech LifeBank Co. Ltd. Shanghai China; ^33^ Shanghai Celevik Biotechnology Co. Ltd. Shanghai China; ^34^ Origincell Co. Ltd. Shanghai China; ^35^ Guangdong Boxi Biotechnology Co. Ltd. Dongguan China; ^36^ Biotechcomer (Xiamen) Technology Co. Ltd. Xiamen China; ^37^ State Key Laboratory of Ophthalmology, Zhongshan ophthalmic Center Sun Yat‐sen University Guangzhou China

## Abstract

‘General requirements for the production of extracellular vesicles derived from human stem cells’ is the first guideline for stem cells derived extracellular vesicles in China, jointly drafted and agreed upon by experts from the Chinese Society for Stem Cell Research. This standard specifies the general requirements, process requirements, packaging and labelling requirements and storage requirements for preparing extracellular vesicles derived from human stem cells, which is applicable to the research and production of extracellular vesicles derived from stem cells. It was originally released by the China Society for Cell Biology on 30 August 2022. We hope that the publication of this guideline will promote institutional establishment, acceptance and execution of proper protocols, and accelerate the international standardisation of extracellular vesicles derived from human stem cells.

## 1 SCOPE

This document specifies the general requirements, process requirements, packaging and labelling requirements and storage requirements for preparing extracellular vesicles derived from human stem cells, and the corresponding confirmation method is described.

This document applies to the research and production of extracellular vesicles derived from stem cells such as human embryonic stem cells, human mesenchymal stem cells and human induced pluripotent stem cells.

## 2 NORMATIVE REFERENCES

The contents in the following documents constitute indispensable clauses of the document through normative references in the text. Among them, dated references, only the version corresponding to this date is applicable to this document; for undated references, the latest version (including all amendments) applies to this document.

T/CSCB 0001 General requirements for stem cells
T/CSCB 0002 Human embryonic stem cells
T/CSCB 0003 Human mesenchymal stem cells
T/CSCB 0005 Human‐induced pluripotent stem cells

## 3 TERMS AND DEFINITIONS

The following terms and definitions apply to this document.

### 3.1 Extracellular vesicle

Particles secreted by cells (spontaneously or induced) with lipid bilayer membrane structure and inability to self‐replicate.

*Note*: Including exosomes, microvesicles, apoptotic bodies, and so on.

### 3.2 Bulk

The culture supernatant of stem cells (collected once or many times) for preparing extracellular vesicles.

## 4 ABBREVIATIONS

The following abbreviations apply to this document.

HBV: Hepatitis B virus
HCV: Hepatitis C virus
HIV: human immunodeficiency virus
HTLV: human T‐lymphotropic virus
EBV: Epstein–Barr virus
HCMV: human cytomegalovirus
TP: treponema pallidum

## 5 GENERAL REQUIREMENTS

5.1 The preparation of extracellular vesicles derived from human stem cells shall comply with the ethical review requirements and biosafety operation specifications of the research centre.*Note*: The preparation process of extracellular vesicles derived from stem cells mainly includes: (a) collection and reception of stem cells; (b) proliferation and induction of stem cells; (c) bulk harvesting; (d) isolation and concentration of extracellular vesicles; and (e) extracellular vesicle harvesting.
5.2 The facilities and environmental conditions for preparing extracellular vesicles derived from human stem cells shall accord with the biosafety requirements, meet the conditions for product quality control and avoid microbial contamination and cross‐contamination.
5.3 According to the preparation process of extracellular vesicles, the primary cell bank, the master cell bank and the working cell bank should be set for the used stem cells, respectively.
*Note 1*: The primary cell bank has uniform storage properties and is suitable for subpackaged cell populations prepared from extracellular vesicles.*Note 2*: The master cell bank stores subpackaged cell populations cultured from primary cell bank cells to a specific doubling level.
*Note 3*: The working cell bank stores subpackage cell populations cultured from the main cell bank cells to a specific doubling level.
5.4 The entire process of preparing extracellular vesicles from stem cell sources shall ensure that the reagents and consumables used, such as culture medium, digestion solution, resuspension solution, elution solution, centrifuge tubes, cryovials, and so on, meet the requirements of sterility, virus‐free, mycoplasma‐free, and low endotoxin. If necessary, validated processes should be employed for sterilisation, and relevant quality assurance procedures should be established.
5.5 After the preparation process of extracellular vesicles derived from stem cells is completed, it should be promptly packaged, labelled, and stored.

## 6 PROCESS REQUIREMENTS

### 6.1 Collection and reception of stem cells

#### 6.1.1 Stem cell collection

Before collecting stem cells for the preparation of extracellular vesicles, ethical approval and informed consent shall be obtained. Meanwhile, the investigation of anamnesis, family history and current health report shall be completed. The detection of infectious disease covers, but is not limited to, HBV, HCV, HIV, HTLV, EBV, HCMV and TP. Other reports, for instance, an official certification of entering and leaving an epidemic hot zone, are also required, if necessary.

#### 6.1.2 Stem cell reception

Ethical approval documents, informed consent, source of cell lines and isolation methods shall be thoroughly checked when receiving stem cells.

### 6.2 Proliferation and induction of stem cells

6.2.1 The proliferation of stem cells shall be carried out in a class A microorganism‐free environment (see Appendix A). Human‐derived stem cells intended for the preparation of extracellular vesicles shall be tested for fungi, bacteria, mycoplasma, HBV, HCV, HIV, HTLV, EBV, HCMV and TP. Stem cells for allogeneic usage shall be free from all the above‐mentioned pathogenic microorganisms, and autologous usage shall be free from fungus, bacteria and mycoplasma contamination. Stem cells containing infectious viruses shall be operated in the dedicated area. The contaminated zone shall be sterilised, and contagion risk must be eliminated after each operation.
6.2.2 Based on the cell types, key quality attributes shall be measured according to T/CSCB 0001, T/CSCB 0002, T/CSCB 0003 and T/CSCB 0005 standards.
6.2.3 Stem cells used for the preparation of extracellular vesicles shall be cultured and proliferated according to the verified standard operating procedures.

### 6.3 Harvest of conditioned medium

6.3.1 Once stem cells reach the stable growth stage, the medium should be refreshed and collected regularly according to cell status.
6.3.2 In the process of harvesting the crude solution (6.3.2), a culture medium with clearly defined components should be used, and substances with unclear components such as animal serum and platelet lysate should be avoided. Unclear substances should not be used during the bulk harvesting stage for extracellular vesicles intended for clinical trial purposes. If reagents with components that are not fully defined, such as animal serum, platelet lysate, and pituitary extract, are used in the culture medium during the bulk harvesting stage, the potential effects caused by these reagents should be considered. Extracellular vesicles should be removed from the relevant reagents in advance.
6.3.3 The bulk shall be packaged separately into autoclaved, virus‐free, mycoplasma‐free and low‐endotoxin containers under sterile conditions. The storage period shall not exceed 48 h at 4°C, and long‐term preservation shall be carried out at −80°C. Importantly, it is better to minimise freeze–thaw cycles.

### 6.4 Isolation and concentration of extracellular vesicles

6.4.1 There are many techniques for isolating and concentrating extracellular vesicles from a conditioned medium, including, but not limited to, differential centrifugation, precipitation with polyethylene glycol, density gradient centrifugation, size exclusion chromatography, ultrafiltration and immunoaffinity chromatography. Notably, heterologous contamination shall be avoided.
6.4.2 Clear process control parameters for the separation and concentration of extracellular vesicles shall be established to ensure the safety, efficacy, and stability of extracellular vesicle preparations. For example, a protocol for isolating extracellular vesicles by differential centrifugation is available in Appendix B.
6.4.3 Microbial and chemical contamination shall be controlled during the entire process of extracellular vesicle isolation and concentration.

### 6.5 Harvest of extracellular vesicles

Verified operating procedures shall be established, and the reagents used shall have clearly defined compositions.

## 7 PACKAGING AND LABELLING

7.1 The vesicles shall be packaged under the same environmental conditions as vesicle isolation with containers made of vesicle‐friendly materials by a validated procedure.
7.2 Each minimum package shall have a unique identification for traceability. The required information is as follows:
  - Product name;
  - Parental cell and generation;
  - Volume and concentration;
  - Manufacturer;
  - Date of production;
  - Patch number;
  - Storage conditions;

## 8 STORAGE

The storage period of stem cell‐derived extracellular vesicles should not exceed 48 h at 4°C. Long‐term preservation should be performed at −80°C, and repeated freeze–thaw cycles should be avoided.

## 9 INFORMATION AND DATA MANAGEMENT

9.1 The whole process of preparation, packaging, labelling and storage of stem cell‐derived extracellular vesicles shall be recorded with a traceable information system. The data retention period is no less than 10 years.
9.2 When genetic‐modified stem cells are used for the preparation of extracellular vesicles, construction details, screening method, exogenous gene sequences, location of integration into the genome, and copy number shall be documented.
9.3 The preparation process of stem cell‐derived extracellular vesicles shall be recorded in detail. The contents of the record are as follows:
  - Patch number;
  - Stem cell attribute, including donor information, cell line, isolation method, and generation;
  - Stem cells quality, including microbial test results, cell morphology, karyotype, cell viability, and protein markers;
  - Manufacturer and equipment;
  - The information of reagents and supplies used in the production, including name, batch number, and lot number;
  - Preparation process and parameters;
  - Environmental parameters, including temperature, humidity, and cleanliness;
  - Manufacture date.
9.4 The packaging and labelling information of stem cell‐derived extracellular vesicles shall be recorded in a timely manner by two staff.
9.5 Each preparation and usage of extracellular vesicles shall be recorded in detail in the ledger, and a file must be created.

## CONFLICT OF INTEREST STATEMENT

No potential conflicts of interest are disclosed.

## Appendix A (DATA): REQUIREMENTS FOR MAIN PARAMETERS OF CLASS A CLEAN ZONE

### A.1

1. The air temperature in the clean operating area shall be 20–24°C.
2. The relative humidity of the air in the clean operating area shall be 45%–60%.
3. Wind speed in the operating area: horizontal wind speed ≥0.54 m/s; vertical wind speed ≥0.36 m/s.
4. Leakage detection of the hePA filter is greater than 99.97%.
5. Illumination: > 300–600 lx.
6. Noise: ≤75 db (dynamic test).

## Appendix B (DATA): EXAMPLE OF PROCEDURES FOR PREPARATION OF EXTRACELLULAR VESICLES FROM HUMAN STEM CELLS

### B.1

Example

**Table:**

File Name	Standard practice for preparation of extracellular vesicles from human stem cells			Code			
Draftsman		Date		Verifier		Date	
Approver		Date		Effective date		Current page/Total pages	1/4
Distribution department							
1. Purpose: To ensure the safe and effective preparation of extracellular vesicles 2. Person in charge: Lab cell culture personnel 3 Scope: Preparation of extracellular vesicles 4. Details: 4.1. Previous preparation 4.1.1. Preparation: Turn on the UV lamp on the purification table for 30 min, then turn off the UV lamp, turn on the fan, and allow air to be blown for 10 min. 4.1.2. Reagents: α‐MEM, normal saline, FBS, serum‐free starvation solution. 4.1.3. Material: 15 mL centrifugal tube, 50 mL centrifugal tube, centrifugal tube rack, T75 cell culture bottle, 10 layer factory, 10 mL graduated pipette, pipette, 10 μL pipette gun, 20 μL pipette gun, 200 μL pipette gun, 500 mL Sterile bottles, lighters and alcohol lamp, reagent bottle shelf, slop, marker pen, cotton ball cylinder, alcohol, watering can, carts. 4.2. Concentrate to harvest When cell growth reaches 80%–90% after 3–4 days, wash the cultured cells twice with 500 mL of phosphate‐buffered saline (PBS) solution. During the washing process, gently shake the cells without allowing them to float. Then, add 500 mL of specialised culture medium (the washing and addition of medium should be completed within 6 min). Place the cells in a cell culture incubator and continue to culture for 48 h with a ventilation device connected. Finally, collect the supernatant of the culture medium. 4.3 Isolation and purification of extracellular vesicles Collect the supernatant of the aforementioned cells. Centrifuge at 300 g for 10 min at 4°C and collect the supernatant after centrifugation. Centrifuge at 200 g for 10 min at 4°C and collect the supernatant after centrifugation. Centrifuge at 10,000 g for 30 min at 4°C and collect the supernatant after centrifugation. Centrifuge at 100,000 g for 70 min at 4°C. Discard the supernatant and collect the pellet. Resuspend the pellet using PBS and centrifuge at 100,000 g for 70 min at 4°C. Collect the pellet. 4.4 Harvesting of extracellular vesicles 4.4.1 Resuspend extracellular vesicles using PBS 4.4.2 Take out 1 mL of the liquid and measure key quality parameters such as protein concentration, particle concentration, and size distribution. 4.5 Packaging and labelling Pack the harvested extracellular vesicles into sterile bottles and label them with key information such as batch number, passage number, and date. 4.6 Store Store the product in a 4°C refrigerator (for no more than 48 h) or in a − 80°C freezer. 4.7. Information storage Records of the preparation of extracellular vesicles from stem cells shall be kept for 10 years.							

